# Editorial: Integrated nutrients management: an approach for sustainable crop production and food security in changing climates

**DOI:** 10.3389/fpls.2023.1288030

**Published:** 2023-10-09

**Authors:** Amanullah, Gabrijel Ondrasek, Abdel Rahman Al-Tawaha

**Affiliations:** ^1^ Department of Agronomy, Faculty of Crop Production Sciences, The University of Agriculture, Peshawar, Pakistan; ^2^ Faculty of Agriculture, The University of Zagreb, Zagreb, Croatia; ^3^ Department of Biological sciences, Al Hussein Bin Talal University Ma’an, Ma’an, Jordan

**Keywords:** integrated nutrients management, chemical fertilizers, organic fertilizers, biofertilizers and nanofertilizers, yield and food security

## Introduction

In an era of shifting climates and evolving agricultural paradigms, the need for sustainable approaches to crop production and food security has become paramount. This Research Topic, titled “Integrated Nutrients Management for Sustainable Crop Production and Food Security in Changing Climates,” presents a collection of pioneering research that addresses the intricate relationship between INM, soil health, and global agricultural sustainability in the context of changing climates (Amanullah, 2017; Amanullah and Fahad, 2018). The issue has been meticulously curated under the editorial guidance of Amanullah, Gabrijel Ondrasek, and Abdel Rahman Al-Tawaha, with the aim of contributing to the advancement of agriculture in a changing world by emphasizing the critical role of INM in increasing crop productivity, reducing fertilizer costs, and solving food security challenges.

## INM and soil health

Integrated Nutrient Management (INM) plays a pivotal role in enhancing soil health, particularly in the context of changing climates. INM's holistic approach involves the synergistic use of organic, chemical, and bio-fertilizers. By maintaining a balanced nutrient profile and fostering beneficial microbial communities in the soil, INM contributes to improved soil structure and fertility (FAO and ITPS, 2016). This is essential for adapting to climate variability, as healthier soils are better equipped to withstand extreme weather events and support sustained crop production (Krasilnikov et al., 2022).

## INM and crop productivity

INM is intrinsically linked to increased crop productivity, a critical aspect of food security in a changing climate. By optimizing nutrient availability to crops, INM ensures that plants receive the essential elements they need for growth and development (Amanullah et al., 2019a; Amanullah et al., 2019b). The judicious use of organic matter, bio-fertilizers, and targeted nutrient applications enhances crop yield and resilience, ultimately leading to higher agricultural productivity. This boost in productivity is vital to meet the growing global demand for food while mitigating the impacts of climate change on crop production.

## INM and costs of chemical fertilizers

One of the primary benefits of INM is its potential to reduce the reliance on expensive chemical fertilizers. With climate change exacerbating resource constraints and increasing fertilizer costs, the adoption of INM practices can be cost-effective for farmers (Amanullah et al., 2020; Amanullah et al., 2021). By incorporating organic materials and bio-fertilizers, INM allows for more efficient nutrient utilization, minimizing the need for excessive chemical inputs. This not only lowers production costs but also promotes sustainable agricultural practices, which are essential in a changing climate (Khan et al., 2022; Imran and Amanullah, 2023).

## INM and food security

Food security is inextricably linked to INM, especially in the face of climate change-induced challenges. INM practices contribute to higher crop yields, ensuring a stable food supply (Khalid et al., 2022). By maintaining soil fertility, INM helps safeguard agricultural productivity against climate-related shocks, such as droughts and floods. Additionally, the sustainable nature of INM reduces the environmental impact of agriculture, preserving ecosystems and safeguarding the long-term availability of food resources (Nadia et al., 2023).

## INM and climate change adaptation

As climate change alters temperature and precipitation patterns, INM emerges as a valuable tool for climate change adaptation in agriculture. INM practices help crops better withstand environmental stressors like heat and water scarcity by enhancing their resilience (Krasilnikov et al., 2022). Moreover, the reduced carbon footprint associated with INM aligns with global efforts to mitigate climate change. By sequestering carbon in soils and reducing greenhouse gas emissions from synthetic fertilizers, INM contributes to a more sustainable and climate-resilient agricultural system (FAO and ITPS, 2016).

These integrated approaches are essential for addressing the complex challenges posed by changing climates and ensuring a resilient and food-secure future.

## Articles and insights

This Research Topic features 12 articles that collectively illuminate various dimensions of INM’s impact on sustainable agriculture. The diverse research contributions delve into vital aspects of agricultural sustainability, exploring novel solutions that extend from nano-technological interventions to organic waste management.

Here are insights from all 12 articles published in the Research Topic on Integrated Nutrient Management (INM), along with their relevance to soil health, crop productivity, lower costs of chemical fertilizers, food security, and climate change, while also including information related to yield and food security:

### Eco-friendly disease management

Khan et al. demonstrate the potential of eco-friendly IONPs synthesized from M. spicata in combatting late blight disease. This research offers an eco-conscious approach to disease control, reducing yield losses and contributing to food security. It also aligns with sustainable agriculture by minimizing chemical pesticide usage and preserving soil health.

### Enhancing manure quality

Holatko et al. explore co-composting cattle manure with biochar and elemental sulfur. This strategy not only enhances soil microbiological characteristics but also improves manure quality. Better-quality manure means improved nutrient content for crops, higher yields, and reduced reliance on synthetic fertilizers, contributing to food security.

### Transitioning to biological fertilizers

Abdo et al. advocate for shifting from chemical fertilizers to biological fertilizers and growth stimulants. Their research underscores the positive impact of these alternatives on crop yield and soil health, promoting higher productivity and food security while reducing environmental impact.

### Alternative fertilization

Dombinov et al. investigate sugarcane bagasse ash (SCBA) as a potential fertilizer for soybeans. SCBA's nutrient-rich composition offers a sustainable alternative to traditional fertilizers, reducing costs, and ensuring a stable yield, contributing to food security.

### Flooding stress tolerance

Yijun et al. explore strategies to enhance flooding stress tolerance in soybean. This research provides insights into mitigating climate-induced challenges, ensuring crop productivity, and safeguarding food security even in adverse environmental conditions.

### Precision agriculture

Li et al. introduce unmanned aerial vehicle (UAV) multispectral imaging for efficient plant nutrient deficiency diagnosis. This technology enhances precision agriculture practices, optimizing nutrient management, and crop yield, ultimately supporting food security.

### Phosphorus and manure integration

Jamal et al. emphasize the integrated use of phosphorus fertilizer and farmyard manure for improving wheat productivity. This approach can reduce reliance on synthetic phosphorus fertilizers, lower production costs, and ensure a stable wheat yield, contributing to food security.

### Nanomaterials in agriculture

Hammerschmiedt et al. explore the effects of graphene oxide and elemental nano-sulfur on soil biological properties and lettuce plant biomass. This research showcases the potential of nanomaterials in sustainable agriculture, with implications for soil health, higher crop productivity, and food security.

### Organic manure alternatives

Lee et al. highlight the value of Hanwoo manure as an organic alternative to chemical fertilizers. This research offers a sustainable pathway to maintaining soil fertility, increasing crop yield, and ensuring food security while reducing the environmental impact.

### Tillage and mowing strategies

Du et al. uncover the impact of tillage methods and mowing time on Cyperus esculentus cultivation. This study provides insights into sustainable practices for specific crops, which are critical for climate-resilient agriculture, higher yields, and food security.

### Improving soil microbial communities

Guo et al. focus on the transformative role of biochar from organic waste in enhancing soil fertility and citrus growth on acid red soil. Improved soil health and fertility contribute to increased crop yield and food security, while the use of organic waste aligns with sustainability.

### Alleviating root rot

Tagele et al. investigate cow dung's potential as a soil amendment to alleviate ginseng root rot. By enhancing soil health and crop resilience, this research supports higher yields and food security in the face of changing climates.

These insights collectively underscore the multidimensional benefits of INM, ranging from improved soil health and higher crop productivity to reduced costs, enhanced food security, and resilience to climate change. Adopting INM practices is a crucial step towards achieving sustainable agriculture and ensuring a stable food supply in a changing world.

## Conclusion and future prospects

This Research Topic underscores the pivotal role of INM in promoting sustainable agriculture, improving soil health, and ensuring global food security in the context of changing climates. The findings collectively support the notion that INM is a fundamental strategy to enhance soil fertility, health, and resilience, which is crucial in the face of climate variability. As research continues to evolve, precision agriculture technologies, genetic diversity preservation, and innovative practices promise to shape a future where sustainable agriculture is the cornerstone of food security, even amidst the challenges posed by changing climates.

## Author contributions

A: Writing – original draft, Writing – review & editing. GO: Writing – review & editing. AA: Writing – review & editing.
